# From Disease to Holiness: Religious-based health remedies of Italian folk medicine (XIX-XX century)

**DOI:** 10.1186/s13002-015-0037-z

**Published:** 2015-06-06

**Authors:** Nelide Romeo, Olivier Gallo, Giuseppe Tagarelli

**Affiliations:** Istituto di Scienze Neurologiche-CNR, Mangone (CS), Italy

**Keywords:** Folk medicine, Religion, Spirituality, Coping

## Abstract

**Background:**

The relationship between spirituality, religion and medicine has been recognized since antiquity. Despite large differences in their history, society, economy and cultures human communities shared a common belief that spirituality and religion played an important role in the healing of diseases.

**Methods:**

The study of religious remedies used by Italian folk medicine in order to treat diseases was based on a review of literature sources compiled between the late nineteenth century and the early to mid twentieth century.

**Results:**

This approach lead to the unearthing of heterogeneous healing methods that have been divided into different categories: Saints, Pilgrimages, Holy Water/Blessed Oil, Blessings, Religious Objects, Contact, Signs, Formulas and The Religious Calendar.

Some of these practices, partly still performed in Italy, are a part of the landscape of the official Catholic Church, others come out of a process of syncretism between the Catholic Religion, the magic world and pre-Christian rituals.

**Conclusions:**

The *vastus corpus* of religious remedies, highlighted in the present work, shows the need for spirituality of the sick and represent a symbolic framework, that works as a filter, mediates, containing the pain that constantly fills everyone’s lives in remote ages even in the third millennium. All of this confirms how important the health-workers know and interpret these existential needs from anthropological and psychological points of view.

## Introduction

The relationship between spirituality, religion and medicine has been recognized since antiquity in human communities living in different geographic areas of the world. Despite large differences in their history, society and economy, cultures from North and South America, The Far East and The Middle East, Africa and The Mediterranean area, shared a common belief that spirituality and religion played an important role in the healing of diseases. Afterwards, above all in Eastern cultures, spiritual and religious components have always been believed to be important factors in maintaining health. Instead, in Western cultures, above all after the Renaissance and the Late Enlightenment, spiritual and religious dimensions have diverged from medicine [1].

Nowadays, bio-medical research around the world, enriched by the contribution of other disciplines such as psychology and anthropology, shows growing interest in the relationship between spirituality, religion and health [2, 3]. In recent years, scientific literature highlighted that spirituality/religiosity can improve recovery from illnesses such as cancer [4], mental disorders [5], cardiovascular diseases, rheumatoid arthritis and head injuries [6].

At the same time it has developed an interesting debate on the definition of spirituality and religiosity. Some authors use the terms spirituality and religiosity as synonyms asserting that the two concepts have common matrix in rites and rituals and so cannot be separated. Others assert that spirituality is associated with finding meaning and purpose in life, transcendence beyond the physical body, and/or experiencing a sense of connectedness with self, others, nature, and/or a power greater than oneself. While, religiosity is associated with human expression of the rites and rituals of a particular faith tradition [7].

Moreover, spirituality/religiosity and culture are inextricably woven together [8]. The rituals, symbols and myths tied to religion can be interpreted as ways of making sense of the world [9]. According to Tarakeshwar, Stanton and Pargament [10], some people who believe in a sacred power view the world through the lens of mythic vocabularies and regulate their lives according to the models and injunctions set forth in their religious traditions.

For all the reasons set out above, cultural competency has become a fashionable term for clinicians too. The clinician can empathize with the lived experience of the patient’s illness, and try to understand the illness as the patient understands, feels, perceives and responds to it. But the large claims about the value of cultural competence for the art of professional care-giving around the world are simply non supported by robust evaluation research showing that systematic attention to culture really improves clinically service. Culture is often made synonymous with ethnicity, nationality and language, so patients of a certain ethnicity are assumed to have a core set of beliefs about illness owing to fixed ethnic traits [11]. Anthropologists, for example, have well documented these kinds of health-related beliefs for several of the ethnic groups in the United States [12]. Cultural differences, however, are not always accompanied by obvious markers such as language and ethnicity. There are people within our own modern society that adopt distinctive values, beliefs and rules for behaviour even if they have the same linguistic, ethnic, religious traits as their fellow-countrymen [13].

An example of this is represented by Italy, which is still today characterized by widespread affiliation to Catholicism (more than 80 % of the population), despite the increase in religious pluralism and undisputed secularization in the customs of population [14]. In Italy, however, to face the moment of illness part of the Italian population still carry out various manifestations of “unofficial” catholic religious expression [15, 16], so like trust in magician to heal (4 % of the population) [17].

The present work aims to unearth the religious remedies used by Italian folk medicine to heal diseases, between the late nineteenth century and the early to mid-twentieth century. The dual propose of this study is to explore how an historical anthropological perspective can provide a different lens through which to explain “unofficial” catholic religious remedies to heal diseases partly still performed in Italy and to interpret their symbolic representations and spiritual meanings.

## Materials and methods

The study of religious remedies used by Italian folk medicine in order to treat diseases was based on a review of literature sources compiled between the late nineteenth century and the early to mid twentieth century.

The literature sources were identified from the online catalogue of the National Library Service of the Italian libraries network, by checking up the following key words: “folk medicine”, “usages and customs”, “folk traditions” and “folk remedies”. The methodology used has led to the consultation of ninety-seven sources (books and journal articles), compiled by anthropologists, physicians, ethnographers, folklorists, and scholars of local history between the late nineteenth century and the early to mid twentieth century.

In Italy, the beginning of this interval represents the period in which the study of folk traditions, including the collection of folk remedies for the cure of diseases, was created and dispersed with well-codified methodological bases [18]. While at the end of the 1950s there was the abandonment of the countryside to the cities so that, for the first time, the Italian population employed in industry exceeded that in agriculture [19]. This phenomenon contributed to the so-called “economic boom” and to the social and cultural transformation of Italy, which went from a predominantly agricultural society to a modern society [20].

Diseases and relative religious remedies used by Italian folk medicine for their care, have been grouped according to International Classification of Diseases (ICD, version 10) endorsed by World Health Organization [21].

## Results

The sources consulted in this study highlighted a *vastus corpus* of religious remedies used to heal various diseases. These numerous sources showed how the interaction between religion and folk medicine has produced at the same time methods that have become part of official religious rites, like the cult of Saints, the use of Holy Water and Blessed Oil, and remedies that highlighted a strong influence between the Catholic religion, magic and pre-Christian practices.

The remedies have been divided into nine different categories: Saints, Pilgrimages, Holy Water/Blessed Oil, Blessings, Religious Objects (Table 1), Contact, Signs, Formulas and The Religious Calendar (Table 2).

**Table 1:** Invocation of Saints, Pilgrimages to sacred places, use of Holy Water/Blessed Oil, Blessings and Sacred Objects used to cure disease

Diseases	Saints	Pilgrimages	Holy Water Blessed Oil	Blessings	Religious Objects
*Certain infectious and parasitic diseases*					
Antrax	Saint Sebastiano [24].				
Cholera	Vergine del Carmine; Saint Gregorio [86]. Saint Rosalia [28]. Saint Gaetano [32].				
Epidemics	Saint Marta [32].				
Erysipelas	Saint John; Saint Agostino [28]. Saint Irene; Saint Elisabetta [32].			Bless the part [45].	
Fever					Wear a scapular [38].
Helminthiasis				Bless the sufferer [46].	
Herpes	Saint Antonio Abate [23].				Wear a scapular [23].
Malaria	Beato Riziero [31]. Saint Domenico; Saint Floriano [23].	Three chapels (Basilicata) [37]. Church of Madonna delle Febbri (Sicily) [28]. Altars of Madonna delle Febbri (Calabria) [87]. Church of Saint Egidio (Abruzzi) where it hangs a pebble behind the front door [24].		Bless the sick [88].	
Plague	Saint Rocco [23, 25, 29, 32, 46, 86]. Saint Osvaldo [23].				Carry in the pocket Saint Giacomo of Compostela’s Agnus Dei [32].
Rabies	Saint Bellino [36]. Saint Domenico da Cucullo [32].	Church of Saint Domenico (Abruzzi) [30]. Church of Saint Vito (Basilicata) [37]. Church of Saint Vito (Sicily) where the sufferer had to exit from another door and not the one they entered in; Church of Saint Vito (Sicily) [28]. Church of Saint Bellino (Veneto) [25].	Holy Water [28].	Bless the sufferer [28].	Wear Saint Vito’s rope [28]. Put Saint Domenico, Saint Stefano, Saint Benigno and Saint Bellino’s key on [38, 43, 46, 47]. Put Papa Leone’s epistle on [89].
Scabies				Bless the part [46].	
Smallpox	Saint Bonosa [32].				
Tinea	Saint Galicano [32].				
Tuberculosis	Saint Giacinto [30]. Saint Teresa [29].	Church of Madonna of Saint Agostino (Latium) [32].			
Typhus				Bless the sick [23].	
Warts			Holy Water [23].		
*Diseases of the blood and blood-forming organs and certain disorders involving the immune mechanism*					
Blood Diseases				Wear a necklace with a small-blessed sack [90].	
*Endocrine, nutritional and metabolic diseases*					
Malnutrition				Bless the sick [24].	
*Mental and behavioural disorders*					
Fear		Church of Saint Vito (Sicily) where the sufferer had to exit from another door and not the one they entered in [28].		Bless the sick after reading the Gospel [91].	
Hysteria		Sanctuary of Canoscio (Umbria) [30]. Church of Saint Gregorio (Abruzzi) [38].		Covering the sufferer with the smoke of incense, blessed olive leaves and salt [92].	
Drunkenness	Patriarca Noè [23]. Saint Quintino [29].				
Madness	Saint Maturino; Saint Aventino [32].	Sanctuary of Saint Ubaldo (Umbria) [30]. Church of Saint Vito (Sicily); Church of Saint Filippo (Sicily) [28].		Wear a blessed shirt [35].	
Mutism	Saint Zaccaria [32].				
*Diseases of the nervous system*					
Chorea		Church of Saint Filippo d’Agira (Sicily); Church of Saint Leonardo (Sicily) where the sick walked three times around the church; Church of Saint Vito (Sicily); Church of Greci (Sicily); Church of Madonna della Nunziata (Sicily) [28].			
Epilepsy	Three Kings [30, 45, 93]. Saint Andrea Avellino; Saint John; Saint Vincenzo Ferrer [28]. Saint Donato [24, 26]. Saint Valentino [23]. Saint Geminiano [36]. Saint Maria in Vade [60].	Sanctuary of Canoscio (Umbria) [30]. Church of Saint Valentino (Veneto) [25]. Church of Saint Geminiano (Emilia Romagna) where the sufferer went under the crypt of Saint [36]. Church of Saint Donato (Abruzzi) where the sufferer was weighed and after offered to the saint the identical weight in grain; Church of Saint Donato (Abruzzi) where they undressed from their old clothes that they left in the church and wore new ones [24]. Church of Saint Vito (Sicily) where the sufferer had to exit from another door and not the one they entered in [28].	Holy Water [28, 32].	Wear a blessed shirt [35]. Drop on the head some blessed wax drips [30]. Tie at the wrists and the ankles some blessed leaves [64]. Covering the sufferer with the smoke of incense, blessed olive leaves and salt [92].	Put Saint Domenico da Cucullo’s medal and keys on; put Holy Spirit’s coin on; brand the skin with Saint Domenico’s iron or key [30]. Wear a necklace with Saint Donato’s silver medal [26]. Put Saint Valentino’s key on [23, 46, 47]. Wear a necklace with a silver blessed key; put Saint Margherita’s little cross on [28]. Put Saint Elena’s and Saint Costantino’s coins on [23].
Headache	Saint Peter; Saint Rita [28]. Saint Augusto [32].	Crypt of Saint Venanzo (Marche) where the sick put their heads on the crypt of the Saint [31]. Oratory of Madonna del Coppo (Emilia Romagna) where the sick went carrying a tile on the head [29].	Holy Water [23].	Bless the head [28].	Put on a lead ring bought in the church of Saint Peter (Sicily) [28]. Tie Saint Augusta’s ribbons around the head [46].
Insomnia	Madonna di mezza montata; Madonna del Buon Riposo [28].				
*Diseases of the eye and adnexa*					
Conjunctivitis				Rubbing a leaf of a blessed palm on the part [24].	
Eye Ailments	Saint Lucia [25, 27–32, 35–37].		Holy Water [28, 30, 42, 43].		
*Diseases of the circulatory system*					
Apoplexy	Saint Andrea Avellino [23, 30, 32, 36].				Put Saint Andrea Avellino’s medal on [30].
Sudden Death					Put Papa Leone’s epistle on [89].
Epistaxis					Put Papa Leone’s epistle on [89].
Gangrene	Saint Pacino [94].				
*Diseases of the respiratory system*					
Sore Throat	Beato Gola [23]. Saint Biagio [25, 28–32].	Altars of Saint Biagio (Friuli) [23]. Crypt of Saint Venanzo (Marche) where the sick put their foreheads on the crypt of the Saint [31].	Blessed Oil [37].	Bless the throat [23, 31]. Wear a blessed shirt [46]. Eat blessed apples [23]. Put the neck between two blessed candles [35].	Wear Saint Biagio’s necklace [28].
Tussis	Saint Tossano [31]. Madonna della Tosse [46].	Church of Madonna della Tosse (Tuscany), coming back from another road and not that they travelled during the outward journey [90].			
*Diseases of the digestive system*					
Hernia	Saint Alfio; Saint Bartolomeo, Saint Giacomo; Saint Corrado [28]. Saint Cataldo [36]. Saint Pantaleone [24].	Saint Calogero’s procession (Sicily) [28]. Church of Madonna del Monte (Apulia) where the sufferer had to exit from another door and not the one they entered in [40].			
Hepatitis	Madonna del giallume [24]. Jesus; Saint Francesco di Paola [28].			Drink a concoction made with an Easter candle, Mary’s candle, hairs and nails of the sick [95].	
Stomachache	Saint Albertino; Saint Santo [30]. Saint Timoteo [36]. Saint Erasmo [32].			Passing a light blessed candle in a glass over the part [96]. Light a blessed candle and place on a coin on the part of the body, then these are covered with an upturned glass [31].	
Stomatitis			Holy Water [38].		
Toothache	Saint Apollonia [23, 25, 27, 28, 30–33].	Church of Saint Apollonia (Sicily) [28].			Touch the part with Saint Domenico da Cucullo’s iron [38].
*Diseases of the skin and subcutaneous tissue*					
Baldness	Saint Peter [28].				
Boils				Pierce the boil with the tip of a leaf of a blessed palm [24].	
Hyperhidrosis			Holy Water [28].		
Milk Crust	Saint Silvestro [46].	Altar of Saint Agnese (Friuli) [23].			
*Diseases of the musculoskeletal system and connective tissue*					
Arthritis	Saint Peter and Saint Paolo [31]. Saint Ventura [30]. Saint Mauro Abate [30, 32].	Church of Saint Ventura (Umbria) where the sick lies down [30].	Blessed Oil [30].	Drop on the part some blessed wax drips [30].	
Dislocations				Drop on the part some blessed wax drips [30].	
Gout	Saint Tommaso [32].	Church of Saint Ventura (Umbria) where the sick lies down [30].			
Sciatica				Bless the part [45].	
Sprains				Light a blessed candle and place on the part and cover with an upturned glass [31].	
*Diseases of the genitourinary system*					
Infertility	Saint Rita [35].		Holy Water [30].	Covering the sufferer with the smoke of incense, blessed olive leaves, crab’s eyes and salt [92].	
To regularize Menstruations	Madonna delle Grazie [30].				
Metrorrhagia	Saint Marta [23].	Church of Madonna della Catena (Sicily) [28].			
Renal Colics	Saint Liborio [28, 30, 32].				
*Pregnancy, childbirth and the puerperium*					
To favour Birth	Saint Anna [25, 29–32, 38, 90]. Saint Libero [46]. Beato Netta [63]. Madonna dei sette dolori; Saint Torello; Madonna delle Grazie; Saint Francesco di Paola [30]. Saint Rita; Saint Leonardo; Saint Nicola; Saint Antonio da Padova [38]. Saint Teodoro [32]	Church near Col San Martino (Veneto) [46]. Church of Saint Teodoro (Latium) [32].		Bless the tummy [32]. Covering the woman in labour with the smoke of blessed olive leaves, blessed candles, sacred images, crab’s eyes, salt, chicken feathers and hairs of the husband [46, 92].	Gird the loins with Saint Francesco’s rope; resting a Crucifix on the lower stomach [46]. Put Papa Leone’s epistle on [89]. Put next to the bed Saint’Anna’s or Madonna’s rose [27, 45, 47, 49]. Wear a necklace with Saint Torello’s medal [30].
Breast Ailments	Saint Agata [23–25, 28]. Saint Joseph [28].				Put Rosary on [28].
Puerperal fevers				Covering the woman in labour with the smoke of incense, blessed olive leaves, crab’s eyes and salt [92].	
To favour the secretion of Maternal Milk	Saint Martino [30]. Saint Agata [27, 30]. Saint Lena [31].	Church of Saint Mamante (Veneto) [46].		Eat blessed food [30].	
*Injury, poisoning and certain other consequences of external causes*					
Snake Bites					Brand the skin with Saint Domenico da Cucullo’s iron [24].
Venomous Bites	Saint Domenico da Cucullo [23, 31, 32]. Saint Giobbe [31]. Saint Paolo [51]. Saint Margherita [30].				Put Papa Leone’s epistle on [89].
Burns	Saint Lorenzo [27–29]				
Contusions				Covering the sufferer with the smoke of a blessed candle [46].	
Cripplings	Saint Eutropio [32].				
Fractures	Saint Mauro [29].				
Wounds	Saint Paolo [25].			Apply blessed fat [31].	
*External causes of morbidity and mortality*					
Drowning	Saint Placido [36].				
Falls	Saint Venanzo [31]. Saint Antonio di Padova [32]. Saint Emarcora [23].				Put Saint Giorgio and Saint Venanzio’s medal on [23, 30].
*Miscellanea*					
All Diseases	Saint Antonio da Padova [29, 35]. Saint Liberata [29]. Saint Cosma and Saint Damiano [28].	Church of Madonna del Pettoruto (Calabria) [97]. Church of Saint Libera (Veneto) [25].	Holy Water [55]. Blessed Oil [46].		Use Madonna di Bibano’s belt [46].
Children Diseases					Put next to the bed the stick of San Francesco di Paola or the hand of Madonna della Consolazione’s statue [98].
Incurable Diseases	Saint Espedito [29]. Saint Joseph [25, 31]. Saint Rita [29].				
Pains		Crypt of Saint Venanzo (Marche) where the sick put their foreheads on [31].

**Table 2:** Contact, signs, formulas and rituals used on certain days of the year to cure disease

Diseases	Contact	Signs	Formulas	Days
*Certain infectious and parasitic diseases*				
Tuberculosis Adenitis		Making crosses using a holy gold ring [30].		
Anthrax			Reciting a formula related to Holy Trinity [37].	
Erysipelas		Making a cross using a metal with the symbol of Solomon engraved on it; making crosses with a gold object, a wedding ring or a silver object [24, 30, 99]. Drawing the sign of Solomon on the thumb [36]. Making three crosses using the fingers [89, 100]. Apply on the part a sheet of paper with a drawing of a circle, four crosses and holy words; applying on the body tracing paper on which is drawned a cross [28].	Reciting a formula rubbing a wool cloth or the feather of a black hen dipped in oil on the part; reciting a formula wetting the part with breast milk and touching it with the point of a scythe; reciting a formula making a cross with a silver coin [38]. Reciting a formula applying leaves of elder in the form of a cross [90]. Reciting a formula related to Mary, God, Saint Nunziata, Saint Croce, Saint Nicola; reciting a formula rubbing a silver coin on the part [55]. Making crosses with the left thumb reciting a formula [89]. Reciting a formula miming nine crosses on the body using a knife [24]. Making crosses with a gold object reciting a formula [32, 45, 64].	
Fevers	Scrape and eat the dust of the church walls or wearing the ground collected close to the Chapel of Madonna del Latte (Abruzzo) [24, 30].		Reciting a formula resting the hands on the forehead [38].	Using coal found during the day of Saint Lorenzo [65]. Drinking an egg at Easter that was produced on Good Friday [30].
Helminthiasis	Hang around the neck a small sack containing different objects including blessed olive leaves [24].	Making crosses on the stomach [28, 49, 62].	Reciting a formula related to Holy Trinity, Mary, Jesus, Saint Cosma, Saint Damiano, Saint Elia, Saint Giobbe, Saint Giuliano, Saint Biagio, Saint Martino, Saint Rosalia, Saint Oronzo, Saint John and Holy Week [24, 28, 40, 52, 55, 89, 90, 98, 101–104]. Reciting a formula shaking the child upside down; Reciting a formula making the patient drink barley water and wetting the mouth with honey; reciting a formula making three times the sign of the cross and massaging with ointment [90]. Reciting a formula massaging the lower stomach [38, 93]. Draw crosses on the umbilicus reciting a formula [56]. Reciting a formula miming the pulling out of worms from the stomach [40].	Rolling the child on the ground on Easter Saturday [31]. Using garlic or wild mint picked on the day of Saint John [23, 30, 45]. Using gentian picked on the day of Holy Cross [60].
Herpes		Applying on the part, nine times, four little leaves of blackberry bush in the form of the cross [100].	Reciting a formula related Saint Antony [28]. Making the sign of the cross using a gold ring and reciting a formula [30].	
Leprosy				Rolling in the meadows during the night of Saint John [59].
Malaria	Drink water of the church of San Mercurio (Sicily); apply a handkerchief that was kept open during the prayer of the Salvatore [28].	Making three crosses using an axe [24, 37]. Miming the sign of cross on the spleen [30, 37]. Apply on the spleen two strips of blu paper in the form of a cross; apply the stalks of a plant called “pastorella” in the form of a cross [24]. Making crosses on the lower stomach [28].	Reciting a formula related Saint Margherita [24].	Drink wine with coal that was found during the day of Saint Lorenzo [28, 65]. Begging for food in three or nine houses during the Christmas night [36, 88].
Mumps		Draw on the part using a feather the sign of Solomon [23, 24, 51, 52]. The sign of Solomon was traced on two hot bricks which are applied to the body [27, 28, 51, 55]. Miming the cross with the thumbs, an axe or using a gold ring [24, 36]. Writing the initials of holy words [24, 37]. The seventh-born of seven sisters made a serious of crosses after putting her thumb in her mouth [47]. On the part of the body you make three crosses using the left hand holding a ladle [55].		
Pediculosis			Reciting a formula related Holy Week and Ascension [28].	
Rabies	Carrying a splinter from the door of the church of San Vito Lo Capo (Sicily) [28].		Reciting a formula related to Saint Antonio [55].	
Scabies				Put on the head the dew collected on the day of Saint John [99]. Diving in the sea or rolling naked in the meadows during the night of Saint John [28, 30, 58, 65, 105]. Diving in the sea or rolling in the meadows during the night of the Ascension [28, 55, 65, 86].
Stomatitis			Reciting a formula dragging the tail of a black cat across the mouth [24].	
Tinea			Making a cross on the head with a gold ring reciting a formula [106].	Submerging the head in two fountains during the day of Saint John [28].
Trombiculosis		Miming with a burning coal the sign of Solomon; Miming a sign of cross with a wedding ring [45].		
Tuberculosis			Reciting a formula applying omentum of calf on the lower stomach and on the back [30].	
Warts	Enter through the church door in which you’ve never been [46].	Miming on the part a cross using a knife [28]. The seventh-born of seven brothers apply on the part two reeds in the form of a cross; burying two wooden stakes in the form of a cross [23].	Reciting a formula related Jesus and Mary [46]. Reciting a formula tieing warts with six strands of straw which are then thrown into a wall [28].	Rubbing an egg on the part produced on the Ascension Day [36].
*Diseases of the blood and blood-forming organs and certain disorders involving the immune mechanism*				
Favism		Sign the sufferer with crosses [95].		Drink fave beans that have been polverized and cooked on Easter Thursday [95].
*Endocrine, nutritional and metabolic diseases*				
Goitre		Making a cross with a wedding ring [30].		Drink an egg produced on Ascension Day [30].
*Mental and behavioural disorders*				
Fear	Drink water in which the medal of Saint Elena and Saint Diego has been immersed [91].	Cutting the hair in the form of the cross [64].		
Madness			Reciting a formula covering the patient with the smoke of incense [28].	
*Diseases of the nervous system*				
Nervous Ailments				Use lavender or drink the decoction of chamomile collected on the night of Saint John [23, 49, 61].
Epilepsy		Making the cross using the tool used to make woollen balls [37].		Use gentian collected on the day of Holy Cross [60].
Headache	Tie a ribbon around the head on the day of Confirmation [30].	Making a cross with the stalk of a plant called “jia petrosa” and put it on the forehead [38]. Put on the head of the patient a bucket of boiling water making crosses on the forehead [24]. Applying on the nape two blessed olive branches in the form of a cross [96].	Reciting a formula related to God, Mary, Saint Joseph, Jesus, Saint Peter, Saint Donato, Saint Silvestro and Saint Flaviano [24, 38, 55, 90]. Reciting a formula putting two black cockerels on the head and on the feet; reciting a formula rubbing the hand on the forehead [38, 101]. Reciting a formula putting a plate of oil on the head; making three crosses while tightening a handkerchief on the head and reciting a formula; reciting a formula making a cross on the head [28].	Put on the head the dew collected on the night of Saint John [23, 24]. Passing over a stream three times on the day of Pentecost [28]. Drink the decoction of bettony collected the night of Saint John; applying the ashes of the first Wednesday of Lent; wearing on the head a crown of flowers on the day of Saint John; wash using the water of seven different springs on Easter Saturday [30]. Passing on the forehead a silver coin wetted with chamomille collected on the night of Saint John [62]. Applying lavender collected on the night of Saint John; drink an egg produced on the Good Friday [23].
Insomnia	Drink the water that rises near the church of Vergine di Santa Maria di Gesù [28].			
*Diseases of the eye and adnexa*				
Eye Ailments	Touch the part with a crystal that came into contact with a relic of Saint Lucia [29]. Wash with the water rises near the Chapel of Saint Lucia [30]. Touch the part with stones collected in the grounds close to the Sanctuary of Santa Maria della Rocca [28].	Making a cross using wedding ring [30].	Reciting a formula breathing on the eyes while chewing rute [90]. Reciting a formula spitting on the ground [45]. Reciting a formula related to Saint Lucia, Saint Joseph, Jesus and Mary [24, 38, 55, 97]. Reciting a formula applying on the eye a wedding ring or a silver object and making signs of cross on the part [24]. Reciting a formula applying a little bough of fennel on the eye; reciting a formula passing on the part the eye of the needle or a gold brooch [27].	Washing using the water of polenta cooked on Easter Saturday [46]. Washing with water on Easter Saturday [23, 36].
Catarats			Reciting a formula making signs of cross [56].	
Conjunctivitis			Reciting a formula lowering down eyelid and moving it from right to left [107].	Using water, which has had roses left in it during the night of Ascension [42].
Pterygium			Reciting a formula applying a small branch of fennel or verbain on the eye passing on it a gold ring. Reciting a formula touching the part with a clove of garlic and making three crosses [28].	
Sty		Miming a cross with a white strand passed through a needle [64]. The sign of the cross must be done by the last of seven children [99]. A twin must make with the tongue the sign of the cross on the eye [91]. Miming signs of crosses [100].		Wetting the eye during Easter Saturday [25].
*Diseases of the circulatory system*				
Epistaxis		Putting two wooden stakes on the nape in the form of the cross [46, 105]. Sign on the forehead the cross using blood from a nosebleed [30]. Putting two stalks in the form of the cross on the head or nape [23, 30, 32, 36, 42, 45]. Sign with a wedding ring the cross on the head [32]. Putting three pieces of straw and three matches in the form of the crosses on the forehead [28, 99]. With the stems of horsetail make a cross on the head [60]. Draw three crosses, one on the forehead and two on the cheeks [23].	Reciting a formula related to God and Jesus [24]	
Gangrene		Sign the part reciting three Ave Maria [94].		
Hemorrhages			Reciting a formula related to Jesus [89].	
Hemorrhoids	Spread the grease of a bell [30].			Use the dew collected on the night of Saint John [24].
*Diseases of the respiratory system*				
Asthma			Reciting a formula massaging the chest [64].	
Cold			Reciting a formula rubbing chamomile oil on the body [38].	
Hiccup		Putting on the back two pieces of straw in the form of the cross [30]. Draw a cross on the Adam’s apple [28].		
Pleurisy			Reciting a formula making a sign of cross [89].	
Pneumonia			Reciting a formula applying leeches to the [38]. Reciting a formula related to Saint Sebastiano [24].	
Rhinitis				Covering the patient with the smoke of pennyroyal collected on the day of Saint Maria Maddalena [28].
Tonsillitis		The sign of Solomon was traced on a heated tile that was applied leaving the sign on the part [108].		
*Diseases of the digestive system*				
Gingivitis				Use the water left in the open air during the Ascension night [32].
Hepatitis		Drink for seven days an egg after drawing a sign of the cross inside with a coin [37].	Reciting a formula related to Saint Costantino [28].	
Hernia	Spread on the part the grease of a bell [36].			On Annunciation or Easter Saturday or Saint John or Holy Cross passing three times the child across a split tree or in a circle created by a branch split in two for the occasion [37, 40, 55, 63–66].
Glandular Inflammations	Rubbing oil taken from a votive lamp [28, 30].	Making crosses with a wedding ring [30].		
Meteorism			Reciting a formula rubbing oil on the lower stomach [28]. Reciting a formula related to Saint Cosma, Saint Damiano, Saint Lorenzo, Saint Apollonia and Saint Leonardo [24, 28].	
Swelling Mouth			Reciting a formula rubbing a key under the tongue [24].	
Ranula			Reciting a formula putting ash under the tongue [28].	
Stomachache	Rubbing oil taken from a votive lamp [30, 31, 46]. Putting on the part the key of the door of Cathedral [97].	Making cross with the thumb [31].	Reciting a formula related to God, Holy Trinity, Mary, Jesus, Saint Peter, Saint Biagio, Saint Giobbe, Saint Martino, Saint Rocco and Holy Week [28, 30, 55, 64, 93, 97, 98, 103, 109]. Reciting a formula rubbing warm oil on the part [24, 55, 109]. Reciting a formula healer walking on the sufferer [28].	Applying on the part a coal found during the day of Saint Lorenzo [49]. Rolling on the ground the day of Easter Saturday [23].
Toothache	Attach and ring a bell using the teeth [30, 38].	Making cross with a wedding ring [30].	Reciting a formula touching the tooth with the root of scotch; reciting a formula touching the tooth with a stalk of wheat [93]. Reciting a formula related to Holy Trinity, Mary, Jesus, Saint Peter and Saint Leonardo [38, 93]. Applying a mush of parsley, oak and acacia reciting a formula [36].	Eat three seeds of grapes during the day of Saint John [36].
*Diseases of the skin and subcutaneous tissue*				
Boils		Drawing with a quill-pen dipped in ink the sign of Solomon [23].		
Acne				Wetting with seawater collected on the night of Saint John [96].
Alopecia				Applying the dew collected on the night of Saint John [23, 24, 30, 36].
Calluses				Stepping barefoot onion on plants during the day of Saint John [30].
Chaps				Applying vinegar used during Christmas dinner [36].
Milk Crust				Wetting the head during Christmas night [58]. Put on the head the dew collected on the night of Saint John [45].
Skin Diseases				Rolling in the meadows during the night of Saint John [31]. Diving in the sea during Christmas night or Ascension night [28, 55].
Skin Eruptions		Drawing the sign of Solomon on the part [46].		Applying the dew collected on the night of Saint John [23, 96].
Hyperhidrosis	Putting the hands on the altar of a church [28].			
Impetigo	Use priest’s saliva [97].			
Lupus			Reciting a formula related Holy Trinity and Mary [24].	
Maculas				Applying the dew collected on the night of Saint John [45]. Rubbing an egg produced on the Ascension day on the part [36].
Wrinkles				Applying the dew collected on the night of Saint John [23].
*Diseases of the musculoskeletal system and connective tissue*				
Arthritis	Rubbing oil taken from a votive lamp [28, 30].	Drawing on the part the sign of Solomon [30, 47].	Reciting a formula bandaging the part with a warm cloth [90].	Applying capon fat used during the Christmas dinner [31]. Use a stone collected on the night of Saint John [96].
Cramps		Miming three signs of crosses on the part [31, 101].	Miming three crosses on the part reciting a formula [28].	
Dislocations		Making crosses on the part [30, 100, 106].	Reciting a formula bandaging the part [89].	
Gout		Drawing on the part the sign of Solomon [47].	Reciting a formula related Jesus, Mary and all the Saints [47].	
Sciatica		Making with oil three signs of crosses on the part [31].	Reciting a formula related to Mary, Saint Sisto and Saint Silvestro [96]. Reciting a formula rubbing hard on the part [24].	
Sprains		Making crosses rubbing oil on the part [28].	Reciting a formula rubbing oil on the part [28]. Reciting a formula related to God, Holy Trinity, Mary, Saint Joseph and Saint John [24].	
*Diseases of the genitourinary system*				
Uterus Ailments				Use the decoction of chamomile collected on the night of Saint John [30]. Use gentian collected on the day of Holy Cross [60].
Renal Colics	Rubbing a stone placed close to the church of Saint Getullio on the back; rubbing the sides on the columns of a church [24]. Rubbing the sides on the walls of a church [38]. Keep in the hand nine walnuts that come from a crib [28].	Making three crosses touching with feet the loins of the sufferer [30].	Reciting a formula while healer sitting astride sufferer [24, 32, 94, 96]. Reciting a formula related to Beato Salvatore [98].	
Impotence				Urinating on a plant of rosemary during the night of Saint John [23].
Infertility				Applying the decoction of various herbs collected on the night of Saint John [30].
To regularize Menstruations				Use lavender collected on the night of Saint John [23].
Metrorrhagia		Put on the lower stomach small pieces of straw in the form of a cross or drawing crosses with ink or soot [23].	Reciting a formula related to Saint Marta [23].	
Wombache			Reciting a formula related to the Holy Trinity and Saint Cipriano [89, 93].	
*Pregnancy, childbirth and the puerperium*				
Abortus	Wearing a glass that came from a church lamp [37].		Reciting a formula related to God [98].	
To favour Birth	Attach and ring a bell using the teeth [52]. Apply on the head a handkerchief that was kept open upon a Mary’s altar [30].		Reciting a formula lighting a candle during Candlemas day [38].	
Colics of Infants			Reciting a formula related to Jesus, Saint Cosma and Saint Damiano [28].	
To eliminate Maternal Milk		Put on the breasts leaves of parsley in the form of a cross [99].		
To favour the secretion of Maternal Milk	Eat the left-overs from table of Capuchins [24, 32]. Wearing the ground collected close to the Chapel of Madonna del Latte [30]. Eat the left-overs from table of Franciscans; wearing chick-peas donated by a priest; drink the water that rises near the churchs of Saint Scolastica, Saint Agata or near the monastery of Franciscans; [24].	Miming three crosses on the shoulders [28].	Reciting a formula related to Saint Agata [24].	
*Injury, poisoning and certain other consequences of external causes*				
Insect Bites		Making crosses with the thumb [110].	Reciting a formula applying a knife on the part [28].	
Venomous Bites			Reciting a formula related to God. Jesus and all the Saints [24, 55].	
Foreign Body in Eyes			Reciting a formula closing the eyes [105].	
Foreign Bodies in Respiratory Tracts				Eat a piece of bread used during Christmas lunch or offered during the day of Saint Biagio [30].
Burns			Reciting a formula drawing a circle on the part [24]. Reciting a formula related to Mary [24, 55].	
Contusions		Miming a sign of cross using a wedding ring or a piece of a plate [28, 46].		Applying butter, pork fat, oil or remains of candle used during Christmas dinner [36].
Fractures				Applying the sticks of cork oak collected on the day of Saint John [111].
Wounds	Drop on the part oil drips previously dropped on a blessed olive leaf [99].	Miming three crosses on the part [38].	Reciting a formula rubbing a cloth dipped in oil and soot on the part [28, 38]. Reciting a formula making with the thumb a sign of cross [89]. Reciting a formula attaching a strand on the part [109]. Reciting a formula related to Jesus, Mary and Saint Lazzaro [24].	Applying butter, oil or pork fat used during Christmas dinner [36]. Use the dew or the lavender collected on the night of Saint John [23, 25]. Rubbing elm’s oil or Saint John’s wort’s oil collected on the day of Saint John [28, 29]. Using oil, which, has had lizards and scorpions left in it and captured during the Ascension day [99]. Use blackberry bush’s oil collected on the day of Ascension day [95].
*External causes of morbidity and mortality*				
Falls				Drink an egg during Ascension day [30].
*Miscellanea*				
All Diseases	Rubbing oil taken from a votive lamp [24, 32].		Reciting a formula related to Jesus and Mary [90].	Use water collected from a public fountain during the Christmas night [97]. Diving in the sea during Ascension day [55, 92]. Using water, which, has had roses left in it during the night of Ascension [55].
Children Diseases	Rubbing oil taken from a votive lamp [46].			
Pains				Use garlic collected on the day of Saint John [59] .

Therefore, because of the impossibility to explain every single remedy, are described, for every category, some remedies that largely favour the comprehension of their use in Italian Folk medicine.

### Saints

To heal themselves from diseases, people entrusted The Holy Spirit, God, Jesus, Mary and the Saints with prayers or acts of devotion (Table 1). The Saint is invoked to heal a specific disease which was chosen because during its martyrdom they were inflicted in the same part of the body that the people have asked to heal [22]. Saint Valentino and Saint Donato, killed by decapitation, were considered the protectors of epileptics [23–26]; Saint Lorenzo, burnt alive, was considered the curer of burns [27–29]; Saint Apollonia to whom, during martyrdom, had her teeth extracted is prayed to by sufferers of toothache [23, 25, 27, 28, 30–33]; Saint Agata who had her breasts cut off, is prayed to by breast feeding mothers [23–25, 27, 28, 30], and so on. Another reason for the choice of the Saint was indicated by its name. The linguistic meaning of the name between the Saint and the disease, according to Italian folklore, was sufficient motivation to ask a Saint to heal a specific disease [34]. For example, Saint Liberata, whose name derives from Latin word “libero”, meaning to release, was invoked for the liberation from all diseases [29]. Similarly, Saint Lucia, from the Latin word “Lux”, meaning light, was considered the protector of the sufferers of eye diseases [25, 27–32, 35–37]. The special healing power was, after all, attributed based on what the Saint did during its life: Saint Biagio was invoked to heal throat ailments [25, 28–32] because he saved a child from suffocation; Saint Anna and Mary were invoked by pregnant mothers because they represent Motherhood [25, 29–32, 38] (Saint Anna being the mother of Mary and Mary being the mother of Jesus); while, Saint Cosma and Saint Damiano, that in their life were doctors, were invoked to heal all diseases [28].

### Pilgrimages

Making a journey to a sacred place was, and still is, a widely used practice in Buddhism, Christianity, Hinduism and Islam. In the Catholic tradition it was thought the disease was a temporal punishment and that healing came from the remission of sin. The pilgrimage was undertaken to obtain plenary indulgence to resolve, hence, more the moral problem than the medical problem [39]. Moreover, very often, the place of pilgrimage was chosen based on “thaumaturgic capacity” of the saint to cure a particular disease, and for this reason, in Italy there was a true network of sacred sites with the function of a “surgery”. The practices put in action when the person arrived at the sacred sites weren’t only religious rites (Table 1). Very often the sufferers used a rite of passage: to cure epilepsy they undressed from their old clothes that they left in the church and wore new ones [24]. Another rite was to weigh the sufferer and offer to the saint the identical weight in grain [24]. To heal Insanity, Hernia and Rabies, the pilgrim had to exit from another door and not the one they entered in [28, 40].

### Holy Water and Blessed Oil

Holy Water and Blessed Oil are among the Sacramentals “instituted for the sanctification of certain ministries of the Church, certain states of life, a great variety of circumstances in Christian life, and the use of many things helpful to man” [41]. In Table 1 are described the remedies used by Italian folk medicine that considered Holy Water and Blessed Oil to be potent symbols of rebirth, capable of eliminating sins and driving away evil, often indicated as the cause of the illness. Humectations of Holy Water were made to cure Stomatitis and Eye Ailments [28, 30, 38, 42, 43]. Holy Water was drunk to treat Rabies and was considered useful to treat Hyperhidrosis submerging the hands in the stoup [28]. Finally, Blessed Oil was wiped to heal Sore Throats and to cure Arthritis [30, 37].

### Blessings

Even today, it is very common in the Roman Catholic Ritual to use various types of Blessings. Priests bless the sick, everyday objects, food, animals, homes, public buildings, etc. [44]. Italian folk medicine used blessings as therapy to heal many diseases (Table 1). For example, to bless the sick or the part of the body concerned, to cure Erysipelas, Typhus and to favour birth without complications [23, 32, 45]; eating blessed food was used to favour the secretion of maternal milk [30] and, even, to heal Sore Throats you needed to wear a blessed shirt [46].

### Religious objects

A large range of objects, associated with healing saints, were used in Italian folk medicine: sacred images, Saint Vito’s rope, Saint Francesco’s stick, etc. Other objects, however, even if they weren’t associated with the saints, were used in its name in to fight certain illnesses (Table 1). The key of Saint Valentine or those of Saint Donato were put in the hand of an epileptic during paroxysm [23, 46, 47] giving the healing power of iron, which was capable of driving away the evil spirits in the sufferer [48]. Another example is the use of the Rose of Jericho, that in Italian folklore was known as the Rose of Mary or Saint Anna [27, 45, 47, 49]. This plant, common in North Africa, Asia Minor, the Middle East, represents the vital cycle that concludes at the start of the dry season, when it folds its branches in a compact spherical mass. In reality, when the rains start the plant rehydrates, the branches open and the growing cycle begins. This characteristic was interpreted by folklore as a magical event in as much as, once watered, was put near the expectant mother with the belief that it would open its branches this would in turn open the uterus ensuring a healthy and incident free birth [50].

### Contact

Italian folk medicine recognised the power of thaumaturgic and all that came from contact with a sacred place or object (Table 2). For example, oil was used in a votive lamp to treat Stomachache [30]; you dragged your sides along the walls of a church to heal Kidney pains [38]; you carried the glass of a church lamp to avoid Abortion [37]; spread the grease of a church bell to cure Haemorrhoids [30]; for Toothache you needed to attach and ring a church bell using your teeth [30, 38]; you carried with you earth collected from a sacred place to combat a Fever [30].

### Signs

Marking on the body of a sick person, was common practice for several illnesses and was done in different ways, sometimes praying or saying mysterious words (Table 2). For example, to heal Mumps, it was drawn directly on the affected part of the body, a cross or the sign of Solomon [23, 24, 51, 52], a symbol with its roots in paganism and in magical symbolism [53], or to write sacred words, for example Jesus, Joseph and Mary [24]. To heal malaria the sign of the cross was mimed on the spleen using the wedding ring or holding a knife or an axe in a clenched fist [28, 30]; to cure Headaches or Epistaxis, the signs were reproduced on the sufferer using a cross made of the stems of certain plants [23, 30, 32, 36, 38, 42, 45]; finally, you needed to trace three times using oil, the sign of the cross into the affected part of the body to treat Sciatica [31].

### Formulas

Using the recital of certain formulas, often accompanied with massage, symbols on the body, the use of ritualistic objects and medicinal herbs or animal parts, religious folklore puts into use a ritual useful for healing (Table 2). The formulas that De Martino defined “historiole” [54] presented a largely homogenous form. The patient began by reciting an episode in the Saint or Jesus or Mary’s life, and finished by begging the disease to disappear.

*Santa Lucia ‘n campa stavia, oru tagliava e argientu facia. Passa Gesù, Giuseppe e Maria, Chi hadi Lucia, chi lacrime jia? Và allu miu ortu e trovi zìpari e finocchi; Cu li lie piedi li chiantai, Cu le mie mani li zappuliai, Esci purvera, esci purata, Esci vena ‘nsanguinentata* [55] (Saint Lucy was in a field, cutting gold and making silver. Jesus, Saint Joseph and Mary passed and asked her? What’s the matter? Why are you crying? Go to my orchard and find figs and fennels; I planted them with my feet, and I hoed them with my hands. Go away dust, go away puss, go away bleeding vein).

In other formulas, more simply the healer invited the illness to leave the sufferer in the name of God, Jesus, Mary, the Holy Spirit or in the name of a saint or even in the name of Christmas or Holy Week.

*Sciuvidia santu/Venerdia santu/Sabatu santu/Pasca santa/Via li vermi/de sta panza* [56] (Holy Thursday, Good Friday, Easter Saturday, Easter Sunday. Go away worms from this stomach).

### Religious calendar

In Table 2, are described the remedies of Italian folk medicine used on certain days of the year dedicated to important Catholic festivals, like Christmas (25th of December), the day of Annunciation (25th of March), Easter (the Sunday following the first full moon after spring equinox), Ascension (40 days after Easter), Pentecost (50 days after Easter), the day of Holy Cross (celebrated on 3rd of May before the reform of the religious calendar in 1970), the day of Saint John the Baptist (24th of June) and that of Saint Lawrence (10th of August). The theory of Frazer suggested that Christianism has integrated, inside its rites many pagan rites that are celebrated based on agricultural and natural cycles. For example, Christmas and the feast day of the birth of Saint John the Baptist can be traced back to pagan festivals that celebrated the time of year in which the daylight began to progressively lengthen (winter solstice) and the period of maximum daylight (summer solstice) that represent the myth of death-rebirth of vegetation making nature fertile (man included) [57]. In this context we can interpret therapies used by folklore medicine for healing various diseases. During Christmas night, for example, people bathed in the sea to fight Skin diseases [55], water taken from public fountains was used to treat Milk Crust in children [58], spreading oil, fat or butter to heal Wounds, Bruises and Arthritis [31, 36]. Similarly during the night of Saint John, sufferers of Leprosy rolled in fields [59], collecting dew to heal Alopecia [23, 24, 30, 36], diving in the sea to cure Scabies [28], medicinal herbs were picked [23, 28, 30, 45, 49, 59–62] because, it was thought their healing virtues grew in these occasions. Finally, a remedy practiced on Annunciation, Easter Saturday, Saint John the Baptist, the Holy Cross to heal hernias in children, consisted of passing the sufferer three times across a split tree or in a circle created by a branch split in two for the occasion [37, 40, 55, 63–66].

## Discussion

Disease represents for mankind one of the most critical and devastating moments in the human life cycle because of its turbulent effect on the individual, giving the sufferer a sense of helplessness, it limits the autonomy and puts in crisis the equilibrium of the whole family group of the sufferer [67].

Previous studies conducted on Italian folk medicine, between the end of XIX and the middle of XX century, to heal malaria [68] and epilepsy [48] have shown, together with a vast body of remedies based on the use of plants and animal parts, a large repertoire of religious practices. The sick entrusted God with prayers or acts of devotion, with the conviction that only God would be able to provide recovery from the above mentioned diseases. In the present work the enormous amount of data has made it necessary to interpret, from the key anthropological point of view in relatively few cases. However, they are considered by the authors to be sufficiently demonstrative of all the corpus of religious remedies used in Italian folk medicine to cure disease shown in Table 1 and Table 2.

To face the moment of crisis, tools were used to identify and symbolize the disease and to cure using coherent therapies based on their own cultural background, which means a set of views, beliefs, values, and attitudes towards life that is transmitted from generation to generation and may be expressed through customs, etiquette, taboos, or rituals [69].

This has determined inside Italian folk tradition a process of syncretism that has brought with it, other than the use of religious remedies that are a part of the landscape of the Catholic Church (begging for the intervention of the thaumaturgic saint, the use of Holy Water and Blessed Oil, pilgrimages and the use of sacred objects), also the use of practices that have come out of a process of contamination between the Catholic religion, the magic world and pre-Christian rituals. The rituals, for example, that call for the use of iron or metallic objects were put into use with the aim of driving away evil, considered as the cause of the disease; hence, passing a child, suffering of hernia, through a split in a tree, or abandoning in church the old clothes and wearing new ones to cure epilepsy, were considered symbols of re-birth which is the abandoning of the old life and acquisition of a new one, free from suffering.

It would be wrong to categorise religious remedies brought to light in this work in the group of the past superstitions. Religious remedies, used in Italian folk medicine between the 19th and the 20th century, form the historical bases of what many people currently perform in Italy, that is turning to prayer or participation in pilgrimages as well as the official medical system of diagnosis and treatment.

An example of engagement with healing prayer are Pentecostal churches [70] that have more of 300,000 Italian devotees [71]. Another excellent example is the cult of Padre Pio whose sanctuary in San Giovanni Rotondo receives million of pilgrims every year [72]. “Devotees of Padre Pio, for his Christ suffering and supernatural visions and stigmata, view the saint as an intimate and invisible friend. So the pilgrimage elicits a distinctive sense of “fellow-feeling”, a sympathetic recognition of another social organism’s humanity, a sense of unity in diversity. Pilgrimage fosters not only a religious experience with a liminal destination, but what Turner calls communitas. Communitas is more than merely sense of “community” – a term which itself is imbued with a geographical sense of common living within a social structure. Rather, occurring in the liminal phase of rituals wherein individuated statuses are suspended as individuals pass from one state to another, or from one structure to another, communitas is social anti-structure, a trascendence of social statuses that serve to order a social community” [73].

On the same basis, part of the Italian population still turns to healers, uses amulets, follows rituals and recites formulas and oaths to treat, for example, “malocchio” (evil eye) that is regarded for mostly physical diseases [15, 16, 74–76]. Moreover, “malocchio” ranks as one of the most searched words in Google Italia in 2014 [77].

Finally, many remedies of Italian folk medicine described in this work (i.e. candles, incense, minerals and Rose of Jerico) are also used by New Agers [50, 78].

There is nothing surprising about it, Garelli [14] in his work highlights that 43 % of Italian catholic population adhere to Catholicism for traditional and cultural reasons or they share its fundamental ideas even if they interpret them in autonomous and subjective way.

The question is if these remedies have had and continue to have a positive impact in facing disease and also if they have some importance from the anthropological point of view. Already, De Martino recognized magical and religious remedies of folk tradition of Southern Italy a protective-psychological value [54]. The authors believe that the vast corpus of religious remedies, highlighted in the present work, can be found inside current coping strategies that patients and relatives adopt to affront the crisis that has hit their personal identity and integrity, kick-starting inner energy that can contribute to the healing process. According to Droogers [79] syncretism seems to occur in an unreflective manner, as a “natural” or better cultural process. As a consequence, people who mix varied religious elements may not do so intentionally and would not necessarily defend or propagate their blended religious practices. Thus, syncretism often serves as a practical mean of solving existential problems.

The spiritual need that the sick shows adopting religious and magical practices, represents a symbolic framework, that works as a filter, mediates, containing the pain that constantly fills everyone’s life in remote ages even in the third millennium [80]. Considering these points, there is the need to add some other explanations more linked to the organic human functions. Many studies report that a silent prayer, meditation or the participation in religious celebration activate the parasympathetic function of the brain, suggesting moreover that these practices can lead to a more active immune response to the pathogenic agents [81, 82].

The theory of PNEI that all our feelings are experienced and psycho-neuro-endocrino-immunologically expressed in the body has been proposed since the late 20th century. Soma and psyche are two aspects of human life and there is a constant interplay between the two [83].

A more detailed analysis on the possible explanation of the functional meaning of suggestion evoked the activation of the limbic-hypotalamic axes, defined as “the locus of information transduction from the neural encoding of the languages of the mind (thoughts, sensations) into the messenger molecules” [84]. It is well known in fact that this system represents a functional connection between the sympathetic control centres (cardiovascular, respiratory, etc.) and the entire endocrine system, with subsequent ability to also influence immune activity [85]. On this ground it is reasonable to hypothesize that the satisfaction of spiritual needs in a stressful condition might support the recovery from individual sufferance *via* the releasing of neuromodulators (i.e. Interleukins) and hormones (Adreno Corticotropic Hormon, Prolactin, etc.) that have been proved to help in the healing process [22].

## Conclusions

The studies concerning the impact of secular forms of spirituality regarding disease, are important currently because they offer to health-workers a key to understanding, from an anthropological and a psychological point of view, which could help to understand, interpret and satisfy the growing need for spirituality the sufferer, deeply rooted in their culture and traditions.

Research such as this, moreover, can give important information about which model of therapeutic belief impacts on modern society and which elements could be integrated into a holistic approach to disease that considers the patients not just as “having symptoms”, as a clinical case but also as an individual that carries a baggage of needs, suffering, hopes and desires.
